# Implementation of mind mapping with problem-based learning in prosthodontics course for Chinese dental students

**DOI:** 10.1186/s12909-023-04479-8

**Published:** 2023-07-25

**Authors:** Yang Yan, Wang Yuehong, Li Kun, Zhou Hongbo, Zhang Hongyu, Yang Yingming, Zhao Zhili

**Affiliations:** 1 Hunan Key Laboratory of Oral Health Research & Xiangya Stomatological Hospital & Xiangya School of Stomatology, Central South University, Changsha, Hunan 410008 China; 2 State Key Laboratory of Oral Diseases, Department of Preventive Dentistry, West China Hospital of Stomatology, National Clinical Research Center for Oral Diseases, Sichuan University, Chengdu, China; 3 Department of Oral and Maxillofacial Surgery, The Second Xiangya Hospital, Central South University, Changsha, Hunan 410010 China; 4 Department of Preventive Dentistry, West China Hospital of Stomatology, Sichuan University, Chengdu, China

**Keywords:** Problem-based learning, Mind mapping, Medical education, Dental students, Prosthodontics

## Abstract

**Background:**

The traditional Chinese dentistry classroom teaching model focuses on the instruction of knowledge details, but less on the frameworks and learners’ motivation. Here, we introduced a combination of mind mapping and PBL instruction (MBL)into the prosthodontics course for Chinese dental students. This study aimed to evaluate the effectiveness and efficiency of MBL in prosthodontics and make observations from the students’ perspectives, based on their response with the learning process.

**Methods:**

We prospectively enrolled 56 fourth-year undergraduates of stomatology, and these participants were randomly allocated into either the combined mind map teaching group (MBL) or the problem-based learning group (PBL) to attend the prosthodontics course. An anonymous questionnaire was also administered to both groups to evaluate the students’ perceptions and experiences, using closed and open-ended items. Data were analyzed using descriptive statistics and thematic analysis.

**Results:**

The students’ responses to closed items indicate their experience in PBL and MBL to be positive, including increased motivation, improved memory of knowledge, enhanced discipline connection and raised teamwork, with fairly higher ratings for the MBL group. However, the tutor-guided competence scores including the memory and framework part, were significantly higher for MBL group than PBL group (two-way ANOVA, *p <* 0.01, *p <* 0.001, respectively). Meanwhile, the self-perceived competence scores including the motivation, framework and teamwork part, were significantly higher for MBL group than PBL group (two-way ANOVA, *p <* 0.01, *p <* 0.001, *p <* 0.05, respectively).

**Conclusion:**

Our findings suggest that MBL teaching approach can help in integration of knowledge structure and enhance clinical reasoning. MBL is an effective and well-organized method in prosthodontics course for dental students.

## Introduction

Prosthodontics is a highly integrated and difficult curriculum based on various clinical dentistry courses. The amount of information that medical students are expected to master is voluminous [1, 2]. In the traditional Chinese undergraduate teaching mode, classroom teaching is conducted in a single mode of lecture, focusing on the teaching of specific knowledge details and paying less attention to the integration of knowledge architecture and framework. Learners often have difficulties in understanding and lack of enthusiasm. Even if they understand the basic knowledge concepts, it is difficult to make vertical or horizontal connections, let alone turn theory into practice [3, 4]. Therefore, how to overcome students’ fear of difficulty, stimulate students’ enthusiasm for learning and cultivate students’ ability to analyze problems has been a challenge for prosthodontics teachers.

Problem-Based Learning (PBL) is a problem-centered, open and exploratory learning method, which is currently one of the mainstream teaching methods in international medical education [5]. PBL advocates student-centered active learning and supplementary comments from the teacher. Students conduct literature review, comprehensive analysis and group discussion based on real-world problems. Meanwhile, the teacher guides, evaluates and summarizes the depth and breadth of the problems based on students’ data preparation and discussion [6]. Compared with the traditional sit-and-listen learning model, PBL is conducive to mobilizing students’ initiative and enthusiasm, and thus improving students’ ability to develop lifelong-learning habits, and to solve complex and open-ended clinical competencies [7, 8].

Mind mapping developed by Tony Buzan is a multisensory tool that uses visuospatial orientation to integrate information [9]. Mind maps can be used as a teaching tool to encourage students to integrate information between disciplines and understand the intra- and inter-relationships between concepts, and consequently helps students to organize and memorize information [10]. The added dimensions of pictures and colors that are unique to mind maps not only facilitates memory, but also benefits students with diverse learning styles. Mind mapping has been confirmed to provide an effective study technique in medical education [11].

In view of the positive role of PBL strategies in guiding students’ learning and the prominent role of mind mapping in concept integration, we combined mind mapping and PBL (MBL) into the teaching of prosthodontics course. Through the carefully designed clinical oriented problems, learners were guided to use mind map to complete basic concepts and clinical cases in small group learning. This study aims to help learners take the initiative to learn, mastering basic theoretical knowledge systematically and achieve the transition from bench to bedside.

## Materials and methods

### Participants

The participants are from two sessions of fourth-year undergraduates of stomatology, one of which is PBL group and the other is MBL group. The inclusion criteria: being a fourth-year undergraduate dental students in Xiangya School of Stomatology, Central South University, and consent to participate the program. The exclusion criteria: foreign students who were unable to use Chinese fluently can participate in this project but not included in the statistics. A total of 56 fourth-year undergraduate dental students were eventually enrolled in the study, with 28 (13 males, 15 females, aged 22 ± 0.64) in the MBL group and 28 (14 males, 14 females, aged 22 ± 0.67) in the PBL group. All participants were randomly divided into 4 subgroups, each subgroup including 7 students.

### Case design

A comprehensive case was introduced into this study. It was based on a real-life scenario of a clinical problem, modifying from previous PBL lesson plans, while incorporating the prosthodontics content and principles of other clinical dental disciplines. To solve this case requires extensive literature review and a summary of the overall knowledge framework structure, which is well suited for PBL and MBL. The solution is not unique and there is no absolutely correct answer.

### Task assignment

Distribute cases and task requirements. The MBL group was trained during a class session to use the mind mapping method, and required to use it as a tool for the presentation of the case solution. Prior to the teamwork, both the PBL and MBL members were asked to complete and submit the case analysis individually, with the MBL group in the form of mind mapping. The PBL group was not required to use the mind map.

### Case solution

Relevant information (literature, textbooks, internet, etc.) was collected by individual. Then, each member of the MBL group presented the case solution by drawing a mind map and the PBL group by text paper. The teacher evaluated the learners’ knowledge acquisition, determined the score based on the work submitted by the students, and provided guidance when necessary, but without undue interference.

### Teamwork

After the individual cases were solved, students were required to work in subgroups and collaborate to form a group opinion and then present their ideas and solutions for the group case through a mind map or text paperwork.

### Presentation

On the day of the lecture, the teacher gave an overview of relevant concepts and latest research progress, and then each group took turns to present the case discussion process and solution, in any form (PowerPoint, video, role play, etc.). After the presentation, other students participated in the discussion, and afterwards, the teacher guided and inspired the students.

### Assessment and summary

Five teachers from the department of prosthodontics acted as judges, to comment and score on each group’s presentation; students assessed each other anonymously within their groups. By reviewing the literature and previous questionnaire, an anonymous Likert type scale questionnaire was designed to examine the effectiveness of the course[12–14]. There were special awards based on the characteristics of each group, regardless of rank.

The teacher explained and analyzed the common problems, controversial and difficult issues, and summarized the key points and difficulties of the course.

### Data collection and analysis

The content validity of the questionnaire was assessed by 3 experts (2 associate professor and 1 attending doctor). Statistical analysis and reliability (Chi-square test) were performed IBM SPSS Statistics 26.0. Descriptive statistics were calculated and were presented as percentages, means and standard deviation (SD). To compare statistically significant differences, the unpaired t-test and Chi-square test were used. P < 0.05 was considered to indicate statistically significant differences.

### Ethical consideration

This study was approved by the Institutional Review Board of the Ethics Committee of Xiangya Stomatology Hospital, Central South University (No. 20,150,004), and informed consent was obtained from all participants.

## Results

### Baseline comparison

A total of 56 fourth year undergraduate dental students participated in two sessions of the course, with 28 in the PBL group and 28 in MBL group. The differences between the two groups on baseline information, such as having participated in PBL or having heard of mind mapping, were not statistically significant (p > 0.05) (Table 1).

**Table 1:** Comparison of the two groups at baseline

Project	PBL group	MBL group	Statistic	*P*-value
Gender (M/F)	14/14	13/15	χ^2^ = 0.072	0.789
Age (X ± SD, years)	22 ± 0.67	22 ± 0.64	T = 0.205	0.838
Have participated in PBL	28	28	NA^*^.	NA^*^.
Have heard of mind mapping	2	4	χ^2^ = 0.747	0.388
Have used mind mapping	0	2	χ^2^ = 1.440	0.149

* NA: Not Applicable

### Student questionnaire responses to closed items

**Fig. 1:**
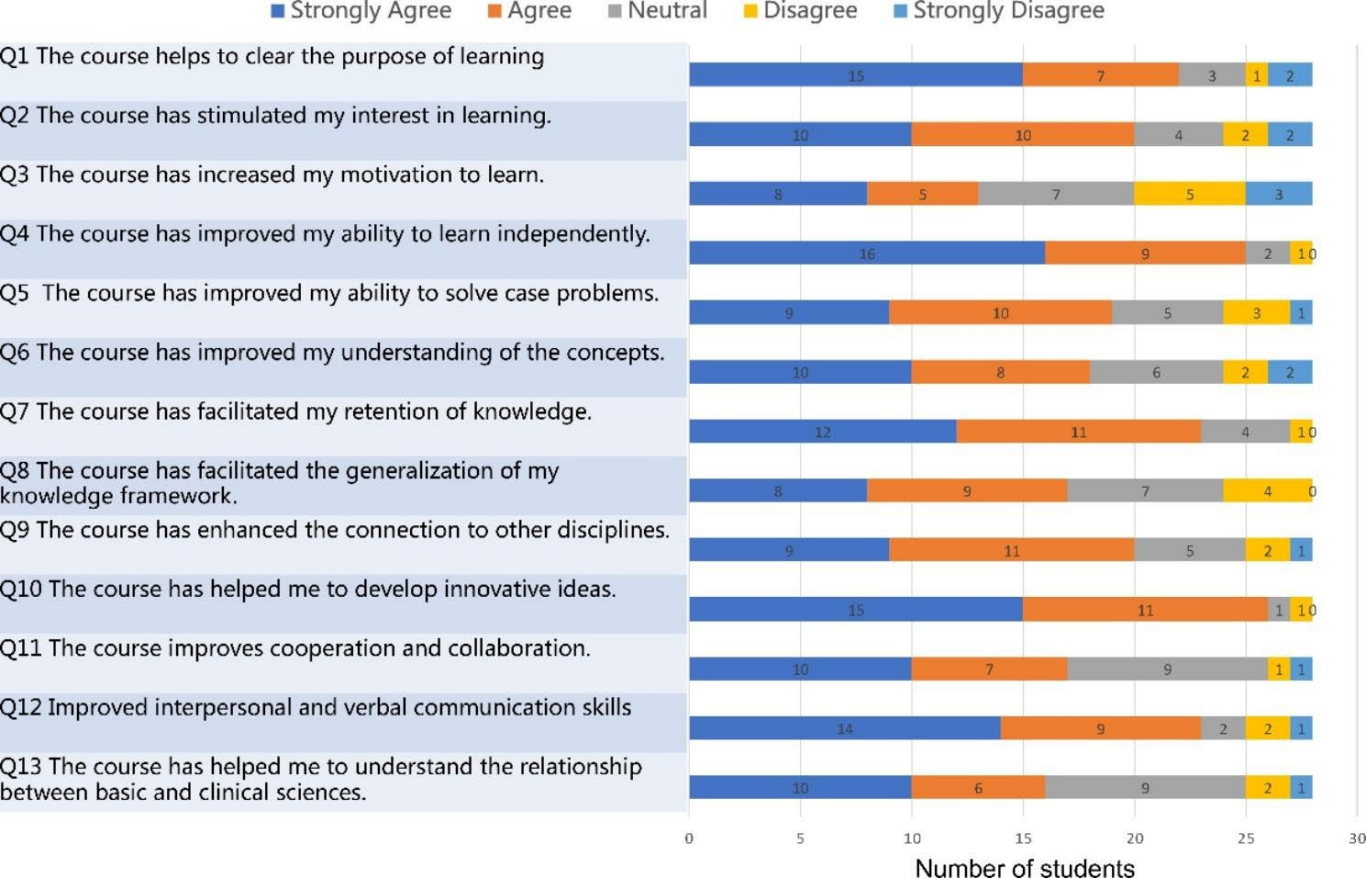
Students’ responses to closed items regarding experience in MBL (N = 28)

**Fig. 2:**
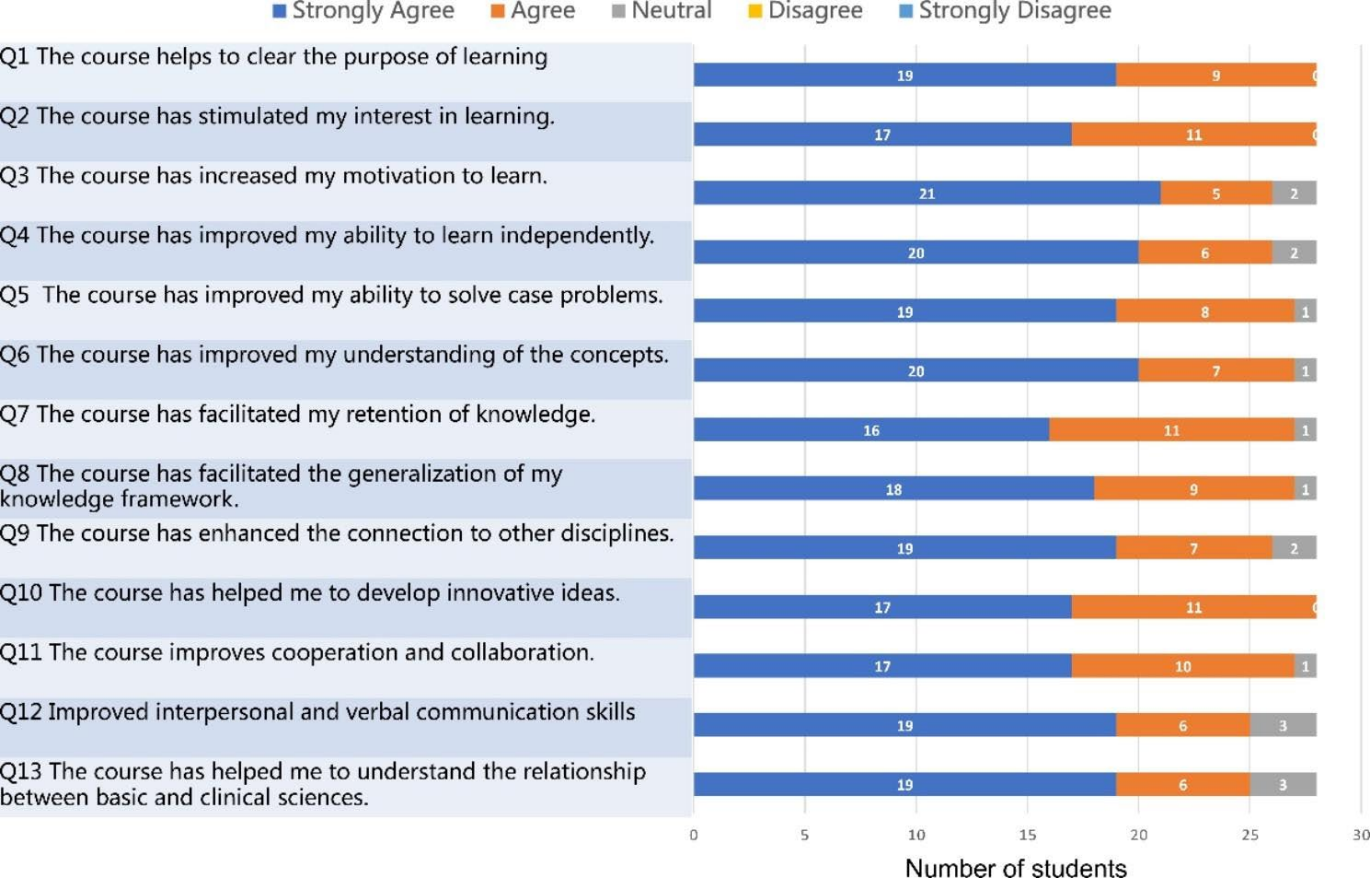
Students’ responses to closed items regarding experience inPBL (N = 28)

The responses of students to closed items regarding their experience in MBL are shown in Fig. 1. Q1-Q3 corresponds to motivation, Q4-Q7 to memory and ability, Q8-Q10 to frame, and Q11-Q13 to teamwork. The responses of students to closed items regarding their experience in PBL are shown in Fig. 2. The distribution of responses across the 5-point Likert scale was similar for both groups of students. Responses to items 1–3 indicated that PBL worked well, with most students giving feedback on increased interest and motivation in the questionnaire. Overwhelmingly, students preferred the mind mapping, with 26 of 28 strongly agreeing or agreeing that in MBL “the course has increased my motivation to learn”, compared to 13 students in PBL (item 3). Feedback was well facilitated in MBL, with 19 of 28 students strongly agreeing that “the course improved my ability to solve case problems,“ compared to 9 students in PBL (item 5). Feedback was also better received in MBL, with 20 of 28 students strongly agreeing that “the course improved my understanding of concepts” compared to 10 students in PBL (item 6). Notably, in MBL, with 18 of 28 students strongly agreed that “the course has facilitated the generalization of my knowledge framework”, compared to 8 students in PBL (item 8), which was consistent with other responses (items 9 and 10) regarding knowledge framework. Importantly, students were generally satisfied with the mind mapping practice within MBL, with 27 of 28 students strongly agreeing or agreeing that “the course improves cooperation and collaboration”, compared to 17 students in PBL (item 11). In MBL, 25 of 28 students strongly agreed or agreed that “the course has helped me to understand the relationship between basic and clinical sciences”, compared to 16 students in PBL (item 13). In general, the comparison of closed-ended questions showed that students were more satisfied with MBL than PBL.

### Student responses to open-ended questions

Student responses to open ended questions regarding the best features and positive features of PBL/MBL are shown in Table 2. The students found positive aspects of their PBL experience to include interesting clinical cases and integration of concept and bench-side practice. In MBL, students reported increased framework integration, knowledge crosstalk, and MBL encouraged interactive learning with students more forthcoming with asking and answering questions.

**Table 2:** Students’ responses to “the best features and positive aspects of the course”

Theme	Examples of students’ comments
Part of Memory	The mind mapping is fantastic, it helps to apply the concepts in the book into the practice. (MBL) The case examples are interesting and relate to the actual situation, which increases the understanding and memory of the knowledge. (MBL) The case design is very impressive and comprehensive, which is helpful to clinical practice. (PBL)
Part of Framework	A very effective way of learning, with a clear logic and framework thinking, to improve the ability to think and deal with problems independently, I hope to propagate. (MBL) The memory of the connection of each knowledge point is strengthened. It is very comprehensive and enhances the horizontal connection of other academic subjects. (MBL)
Part of Teamwork	I would like to give a higher rating than a 100% for the improvement of the creative skills. (MBL) It was useful to simulate the communication between doctors in different departments in the clinic through role play. (PBL)

Responses to open ended questions regarding the most difficult features of course and suggestions are illustrated in Table 3. Both groups commented a bit complicated clinical setting and kind of difficulty for critical thinking demand; and felt more clinical experience would be helpful to reach decisions on diagnosis and management. For the MBL group, they suggested further popularization of MBL to other teaching courses. They also reported they would readily utilize mind mapping as a learning tool in their future studies.

**Table 3:** Student in MBL group responses to “the most difficult features of course and suggestions for improvement”

Theme	Examples of students’ comments
Part of MBL	The topic was challenging, but after drawing the mind map, it was not so difficult and interesting. I will continue to use mind mapping as a way of learning in the future. It does not seem to be carried out in other sessions, so it is recommended to continue to promote it. It is recommended to continue the case discussion with mind maps. The protocol design often does not meet clinical needs and is not mature enough to continue using this format, but try other topics or cases.
Part of PBL	The topic is too difficult! Whether we should experience the clinic before doing this kind of case, otherwise it is difficult to choose the best option. As a student, I think it is still quite difficult to make a comprehensive diagnosis and treatment of this complex case, and clinical knowledge is very lacking. In groups, each group member has a different level of participation.

### The comparison of tutor-guided competence scores between the PBL and MBL groups

**Fig. 3:**
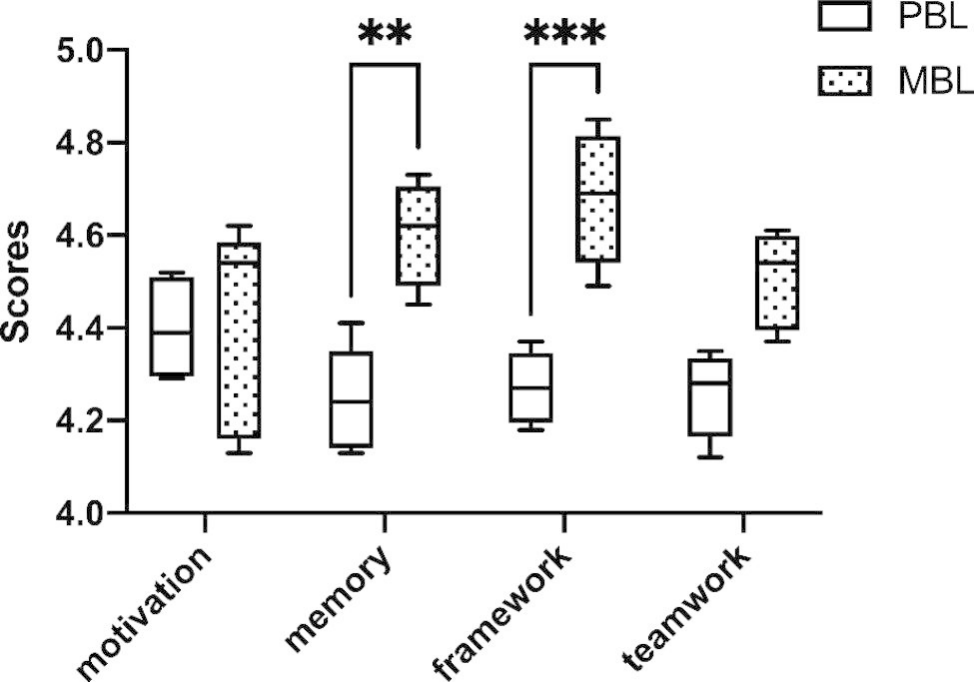
The comparison of tutor-guided competence scores between the PBL and MBL groups. (** means *p <* 0.01, *** means *p <* 0.001)

We compared the PBL and MBL groups’ tutor-guided competence scores (shown in Fig. 3). In the PBL group, the mean competence scores of motivation, memory, framework and teamwork were 4.40 ± 0.11, 4.24 ± 0.11, 4.27 ± 0.08, 4.26 ± 0.09, respectively. Meanwhile, for the MBL group, they were 4.41 ± 0.23, 4.60 ± 0.11, and 4.68 ± 0.14, 4.51 ± 0.11, respectively. It is notable that the MBL group’s framework competence scores were significantly higher than the PBL group’s (*p <* 0.001). Similarly, the MBL group’s memory competence score was significantly higher than the PBL group (*p <* 0.01). Furthermore, the motivation competence scores and teamwork competence scores in MBL group were higher than PBL group, which was not statistically significant (*p* = 0.99 and *p* = 0.12, respectively).

### The comparison of self-perceived competence scores between the PBL and MBL groups

**Fig. 4:**
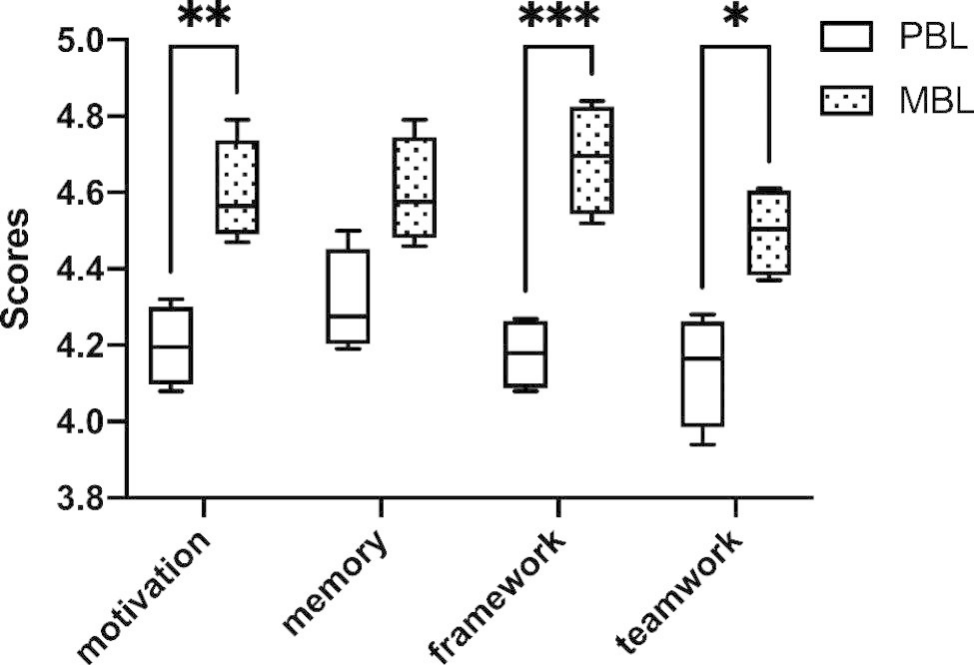
The comparison of self-perceived competence scores between the PBL and MBL groups. (* means *p* < 0.05, ** means *p* < 0.01, *** means *p* < 0.001)

In order to further evaluate the students’ learning experiences, we conducted a self-perceived competence score (shown in Fig. 4). For the PBL group, the mean competence scores of motivations, memory, framework and teamwork were 4.20 ± 0.10, 4.31 ± 0.14, 4.18 ± 0.10, 4.14 ± 0.15, respectively. Meanwhile, for the MBL group, they were 4.60 ± 0.1 4, 4.60 ± 0.14, and 4.69 ± 0.15, 4.50 ± 0.12, respectively. The self-perceived scores of motivations and framework were significantly higher for the MBL group than for the PBL group (*p <* 0.01 and *p <* 0.001 respectively). For the MBL group, the scores for memory were higher than those in the PBL group; however, there was no statistically significant difference between the two groups.

## Discussion

### Literature review

PBL originated in medical education in the 1950s and was first proposed by Barrows at McMaster University in Canada, and has now become one of the common teaching methods in international medical education [15, 16]. The implementation of PBL teaching, which places students in the midst of clinical problems, helps to mobilize students’ motivation and initiative, guide them to think independently, retrieve information, promote the application of what they have learnt, link theory to practice and develop their ability to apply their knowledge in a comprehensive manner [7, 17]. However, although the PBL teaching mode has many advantages, it still faces certain obstacles in the teaching of undergraduate students [18, 19]. Due to the influence of the long-term traditional education model and examination-based education, students are used to “duck-fill” teaching, and their ability to acquire knowledge tends to be passive and fragmented, and we found that students’ problem-solving ability is still one-sided in the past PBL teaching of prosthodontics. Therefore, it is a challenge for us to break the conventional thinking and change the way of thinking of students.

The application of modern technology in teaching activities is an important tool and measure to improve the quality of undergraduate teaching [20]. As an organizational thinking tool, mind maps organize and represent the relationship between knowledge and knowledge by drawing diagrams, enabling learners to intuitively grasp the entire knowledge framework and system, enhancing memory and logical thinking skills [11, 21]. Mind maps have been used to graphically display participants’ ideas and recommendations from international dental education conferences that have prompted discussions about interprofessional collaboration in patient care [9]. Dental students have responded favorably to the use of mind mapping, which can benefit students with different learning styles and help instructors determine the level of conceptualization of a topic [22–24]. In the process of PBL teaching in prosthodontics, we have tried to introduce mind mapping to solve the problems encountered in previous teaching.

### Question design

The questions are designed according to the characteristics of the participants and subject features. The subject has completed all the basic and clinical disciplines and has the ability to analyze and judge common diseases in all dental disciplines, and is about to enter clinical practice after years of theoretical study. Therefore, the lecturer at this time, based on the requirements of the Prosthodontics syllabus, uses clinical cases that incorporate multidisciplinary content and clinical real-life scenarios as an entry point, integrates all the main points of knowledge in Prosthodontics, as well as the principle knowledge of other dental clinical disciplines related to Prosthodontics, stimulates students to conduct extensive literature and internet resource searches, and develops students’ ability to grasp the knowledge framework, clinical thinking skills, and problem-solving skills. Considering the fairness of the knowledge received by the students, PBL and MBL groups were set in two separate grades for comparison, while no teaching method control was set in the same grade.

### Task implementation

Tasks are issued on the basis of the students’ basic theoretical knowledge of the subject in order to reduce the sense of confusion in the learning process. Students are encouraged to collect information in multiple ways (literature search, textbooks, internet, etc.), form group ideas through self-study and group discussion and present them in a variety of ways (Power Point, video, role play, etc.) to guide students to bring their individual strengths into play, promote the participation of each student and fully stimulate their creative and teamwork abilities.

### Judges’ comments

Either the PBL or the MBL teaching method, requires teachers to change their role from leader to organizer and guide, and also places higher demands on their quality. In addition to being proficient in theoretical knowledge, teachers have to improve themselves in terms of teaching ideas, teaching skills and mastery of modern technology. In order to drive students’ thinking in a multi-dimensional and multi-level way, all clinical teachers in the teaching and research department participated in the lectures and acted as assessors.

### Evaluation and assessment

In the construction of the teaching evaluation system, the teaching department attaches importance to the assessment of students’ ability and has moderately increased the proportion of PBL. In the self-study stage, teachers review the mind maps submitted by students to examine their ability to analyze and solve problems independently and to avoid speculation. In the team presentation stage, judges give case discussion grades based on each group’s mastery of basic knowledge, problem solving, sense of innovation and group cooperation. At the same time, the teaching effectiveness is anonymously evaluated by the students. Finally, the lecturer uses a symposium to share with students the shortcomings and suggestions of the course. The diversity of the evaluation system in order to improve the credibility of the assessment and objectively reflect the teaching effect has to a certain extent facilitated the implementation of the teaching reform, thus promoting more strongly the quality education of students.

Through anonymous questionnaires and competency scores, it was found that the introduction of mind mapping tools in PBL teaching activities had significant advantages in enhancing students’ memory of knowledge and mastery of the curriculum framework. Besides, self-perceived competency scores indicated that the flexible and varied presentation format stimulated students’ learning interest and initiative; the visual and clear image format enhanced students’ communication and teamwork. Also, the clinical cases integrated almost of the knowledge points of prosthodontics with multidisciplinary content and clinical scenarios, stimulating and facilitating students’ ability to grasp the disciplinary framework, make multidisciplinary connections, and solve clinical problems.

After the initial exploration of this teaching reform, we found that the combination of mind mapping and PBL teaching method greatly promoted the students’ ability to master the subject framework, the horizontal connection between multiple disciplines and the ability to solve clinical problems, so that they were given more space for self-development and were also more interested in the subject knowledge of prosthodontics and willing to communicate with the teachers more often. The implementation of teaching activities needs to be integrated with the students’ reality and with the characteristics of the discipline of prosthodontics, to continuously improve the quality of teachers and to reform the teaching mode and evaluation system in order to better meet the needs of higher medical personnel training [6].

## Conclusions

This study identified that combination of mind mapping and PBL teaching can help students with integrated concept mapping and achieve a more complete knowledge structure, which is crucial as students move toward clinical immersion. In conclusion, MBL is an effective and well-organized method for Chinese dental students on the prosthodontics curriculum.

## Data Availability

Datasets supporting the conclusions of this article are included within the article. Additional data at the level of individual students is not available as per confidentiality agreements approved by the Institutional Review Board of the Ethics Committee of Xiangya Stomatology Hospital, Central South University, but can be available from the corresponding author upon reasonable request.

## Abbreviations

PBL: Problem-based learning
MBL: Combination of mind map and problem-based learning
